# Forecasting the regional distribution and sufficiency of physicians in Japan with a coupled system dynamics—geographic information system model

**DOI:** 10.1186/s12960-017-0238-8

**Published:** 2017-09-12

**Authors:** Tomoki Ishikawa, Kensuke Fujiwara, Hisateru Ohba, Teppei Suzuki, Katsuhiko Ogasawara

**Affiliations:** 1 Graduate School of Health Sciences, Hokkaido University, N12W5, Kita-ku, Sapporo, 060-0812 Japan; 2 Department of Radiological Technology, Faculty of Health Sciences, Hokkaido University of Science, 7-Jo 15-4-1, Maeda, Teine-ku, Sapporo, 006-8585 Japan; 3 Faculty of Health Sciences, Hokkaido University, N12W5, Kita-ku, Sapporo, 060-0812 Japan

**Keywords:** Forecasting, Physicians, Physician shortage, Maldistribution, System dynamics

## Abstract

**Background:**

In Japan, the shortage of physicians has been recognized as a major medical issue. In our previous study, we reported that the absolute shortage will be resolved in the long term, but maldistribution among specialties will persist. To address regional shortage, several Japanese medical schools increased existing quota and established “regional quotas.” This study aims to assist policy makers in designing effective policies; we built a model for forecasting physician numbers by region to evaluate future physician supply–demand balances.

**Methods:**

For our case study, we selected Hokkaido Prefecture in Japan, a region displaying disparities in healthcare services availability between urban and rural areas. We combined a system dynamics (SD) model with geographic information system (GIS) technology to analyze the dynamic change in spatial distribution of indicators. For Hokkaido overall and for each secondary medical service area (SMSA) within the prefecture, we analyzed the total number of practicing physicians. For evaluating absolute shortage and maldistribution, we calculated sufficiency levels and Gini coefficient. Our study covered the period 2010–2030 in 5-year increments.

**Results:**

According to our forecast, physician shortage in Hokkaido Prefecture will largely be resolved by 2020. Based on current policies, we forecast that four SMSAs in Hokkaido will continue to experience physician shortages past that date, but only one SMSA would still be understaffed in 2030.

**Conclusion:**

The results show the possibility that diminishing imbalances between SMSAs would not necessarily mean that regional maldistribution would be eliminated, as seen from the sufficiency levels of the various SMSAs. Urgent steps should be taken to place doctors in areas where our forecasting model predicts that physician shortages could occur in the future.

## Background

Supply and demand imbalance in human resources for healthcare is a global issue [1]. In particular, the shortage of physicians is a common problem not only in developed nations but also in developing countries [2]. In Japan, the shortage of physicians has been recognized as a major medical issue. The number of physicians per 1000 residents in Japan is 0.93 smaller than the average for member countries in the Organization for Economic Cooperation and Development (OECD) [3]. Japan stands 63rd among members of the World Health Organization (WHO) in the number of physicians per residents [4].

In our previous study, we reported forecasting the absolute and relative shortage of physicians in Japan by using system dynamics modeling [5]. We showed two key results. First, the absolute shortage will continue until 2026. Second, the shortage of obstetrics/gynecology (OB/GYN) specialists would continue for a period subject to analysis. These results suggest that the absolute shortage will be resolved in the long term, but maldistribution among specialties will persist. According to the biennial “Survey of Doctors, Dentists, and Pharmacists,” the number of physicians in Japan as a whole is growing steadily over time; the growth rate from 2000 to 2014 was 18.1%. In the same way, the number of physicians per 1000 residents is growing and was 2.34 in 2014. However, the Ministry of Health, Labour and Welfare (MHLW) has reported that inadequate numbers of physicians result from absolute shortage and maldistribution by region or specialty [6]. The number of physicians per 1000 residents varies, from the highest (Kyoto Prefecture) at 3.08 to the lowest (Saitama Prefecture) at 1.53 [7]. The differences could be considered an aspect of regional maldistribution. In this way, while the total number of physicians has been increasing, regional maldistribution still remains.

Unlike countries such as the UK, there is no system to allocate physicians in Japan. Physicians in private practice can choose their preferred location. The principle of freedom to open their own practice, called the “free medical practitioner system,” is considered one of the factors contributing to regional maldistribution [8]. In addition, medical school graduates can select their specialty and location based on their preferences. This is also considered a factor in regional and specialty maldistribution.

To address regional shortage and to secure the number of doctors in underserved areas, several Japanese medical schools established “regional quotas” in 1997 [1]. The regional quota system promotes practice in specific areas by offering scholarships with a term-defined practice requirement. In 1997, two medical schools adopted this regional quota system, and 11 students entered the program. In 2015, 70 of 79 Japanese medical schools offered regional quota programs, admitting 1541 students (approximately 18.9% of all medical students). The Ministry of Education, Culture, Sports, Science and Technology (MEXT) reported in 2015 that graduates of the regional quota system had higher retention rates (83.3%) than their peers (45.3%) [9]. Consequently, the Japanese government expected that the regional quota system would decrease regional maldistribution.

### Methodology for analysis of health care workforce

Accurate forecasting of the workforce supply is essential for evidence-based health workforce policies. Some study has attempted to develop methodology using simulation approach to forecast medical workforce. Forecasting methods include the demand-based model and supply-based model. [10]. The former is a forecasting method focusing on future healthcare service utilization [11, 12]; the latter is a method focusing on the production of services or the number of trainees and retired [13].

Hagopian et al. produced a demand-driven staffing model based on treatment protocols, and projected the need for health workers for HIV/AIDS care at three scenarios depending on numbers of patients [11]. Tjoa et al. developed a model to forecast the size of the public sector health workforce in Zambia over the next 10 years and suggested that meeting the minimum need for healthcare in the future would require an increase in health training school enrolment [12]. Takata et al. showed possibility bringing about a doctor surplus in the long run by simulation modeling of the Japanese the physician number, considering increase in the student quota [13]. These analyses have several limitations, one of which is that they cannot consider dynamic factors in the model. In recent years, system dynamics modeling is widely used as being a method suitable for forecasting of human resources for health, because it permits the inclusion of a dynamic factor in the models [5, 14–17].

Barber et al. conducted [16] demand/need simulation for 43 medical specialists using system dynamics model. Ansah et al. [17] used system dynamics methodology and model to project outlook of supply need for eye care in Singapore and the number of eye disease and the demand eye care services as well as workforce requirements will rise. In Japan, we have developed forecasting model to evaluate at the national level supply [5]. In addition, time trends in number and regional distribution of physicians has been analyzed. Toyabe et al. [18] used Gini coefficient, Atkinson index, and Theil index as measures for maldistribution of physicians to population and the regional maldistribution will worsen after the new postgraduate internship system starts. Thus, analysis of regional maldistribution is necessary not only time trends analysis but also future [5].

### Purpose of this paper

Japanese government policy on medical education would require periodic reassessment to accommodate the future needs of each area. To help optimize policy, supply–demand balances forecasting is a crucial step to assess the adequacy of the current strategy. At present, however, the relationship between these new measures and future geographic distribution of physicians is unknown. In our previous study, we determined the importance of forecasting absolute numbers and analyzing regional needs.

This study aims to assist policy makers in designing effective policies, with a data-driven evaluation of regional inequities and future physician numbers in each area. We built a model for forecasting physician numbers by region to evaluate future physician supply–demand balances.

## Methods

### Study area

**Fig. 1:**
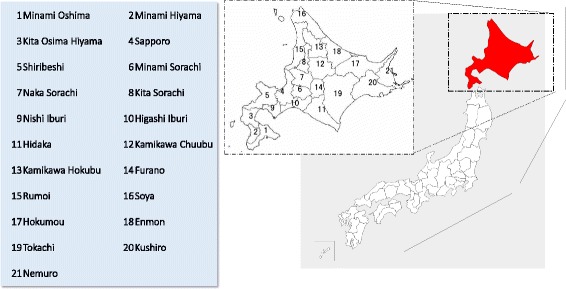
Location of Hokkaido within Japan; each secondary medical service area (SMSA) in Hokkaido

We selected Hokkaido Prefecture (Fig. 1) as our case study. Hokkaido is a large land area, covering 83 000 km^2^ or 22% of the total area of Japan [19]. Optimizing the distribution of healthcare resources must consider the transportation methods available to the local population to access healthcare facilities. Hokkaido’s population is largely concentrated in Sapporo, while depopulation is progressing in remote areas [20]. This trend has led to disparities in social services among the various local economies.

**Fig. 2:**
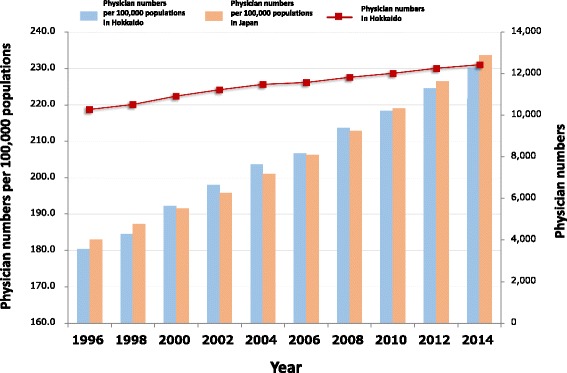
Physician numbers in Hokkaido and Japan

As is the case for Japan as a whole, physician supply and demand in Hokkaido is characterized by both a shortage in absolute numbers and an unequal distribution among geographic areas and medical specialties. Figure 2 shows that the number of physicians in Hokkaido is growing concurrently with the number of physicians nationwide. However, the number of physicians per 100 000 residents in Hokkaido is far lower than the number for Japan as a whole. Hence, there is an absolute and a regional physician shortage in Hokkaido; policies to remedy the maldistribution would benefit from estimating future needs.

### Study design

This study applied the STELLA system dynamics (SD) model software by Isee Systems to evaluate the future supply–demand balance in healthcare by region (Isee Systems Inc., Lebanon, NH, USA) [21]. We employed ArcGIS software (ESRI, Redlands, CA, USA) as geographic information system (GIS) technology, to analyze the dynamic change of spatial distribution of indicators contained in the SD model [22]. GIS software tools enable researchers to analyze and visualize spatial information [23–25]. The integration of geographically referenced data from a variety of agencies concerned with health issues enables researchers to visualize trends and relationships over time and space to monitor the influence of government policies, such as those aimed at reducing health inequalities. However, few studies to date have focused on dynamic forecasting by using GIS. In recent years, a combination of SD modeling and GIS has been proposed as a way to fulfill mutual feedback modeling in time and space [26, 27]. SD models can extend the spatial analysis functions of GIS to realize both dynamic simulation and trend prediction of system behavior.

This study employed a “loosely coupled SD-GIS” assessment method to identify the change in the supply–demand balance over both area and time. The procedure follows four steps. The first step is data collection. The second step involves designing a causal loop and stock-flow diagram and constructing forecasting models. A causal loop diagram identifies the structures and interaction of feedback loops, and consists of variables for possible causal links. The third step is to analyze dynamic change and spatial distribution of indicators by using SD and GIS technologies. In this step, SD was employed as tool of forecasting the number of physicians in each area, GIS was employed for calculation of the number of physicians per 100 000 residents based on population statistic data by area. The final step is to evaluate dynamic change and spatial distribution by the results of the forecast.

For Hokkaido overall and for each secondary medical service area (SMSA), divided geographical unit for medical care planning, we analyzed the total number of all practicing physicians. Our study covered the period 2010–2030 in 5-year increments. We obtained data on the number of physicians from the national surveys of physicians, dentists, and pharmacists conducted from 1972 to 2008 by the MHLW. The retirement rate was calculated by trend data considering the age–sex pyramids of the medical population. Data sources are summarized in Table 1.

**Table 1:** Data sources of model input

Variable name	Source
Parameter value	
General quota in Hokkaido	MEXT
Regional quota	Bulletin about scheme of increases medical school quota by MEXT
Rates of passing national exam	Announcement about national examination for medical doctors
Rate of matching residency with hospital	Japan residency matching program conference
Rate of students who go on to graduate school	The school basic survey carried out by the MEXT
Choosing rate of non-clinical occupation	The school basic survey carried out by the MEXT
Rate of staying in Hokkaido	Trend data for the numbers of physicians reported by the MHLW
Choosing rate other prefecture	Trend data for the numbers of physicians reported by the MHLW
Rate of inflow to Hokkaido from other area	Trend data for the numbers of physicians reported by the MHLW
Rate of outflow from Hokkaido to other area	Trend data for the numbers of physicians reported by the MHLW
Option rates each SMSA as occupational place	Trend data for the numbers of physicians reported by the MHLW
Require number of physician in Hokkaido	National Survey of the required number of physicians in hospitals by MHLW
Require number of physicians in each SMSA	National Survey of the required number of physicians in hospitals by MHLW
Rates of retirement (include death, change of occupation)	Trend data for the numbers of physicians reported by the MHLW
Stock value	
Medical students	MEXT
Graduate students number	MEXT
Resident in Hokkaido	Japan residency matching program conference
The number of physician in Hokkaido	MHLW
The number of physician in SMSA	MHLW
Population projection in Japan and Hokkaido	National Institute of Population and Social Security Research

### Data

#### Constructing a forecasting model using system dynamics

**Fig. 3:**
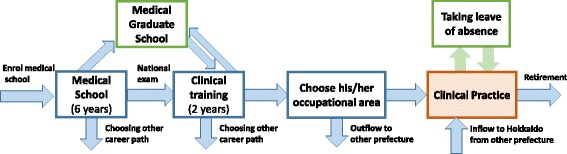
Conceptual scheme of the Japanese physician career path

Using the SD concept, we designed a flow chart showing the physician career paths illustrated in Fig. 3 for the physician numbers in our study. We input values for the stock, flow, and converter categories using the statistical data on physician career paths, and established the associated variables.

#### The validity of the SD model

The validity of model forecasts should always be tested. As a necessary step in SD methodology to validate the model, historical and simulated data should be compared for specific years. We examined the simulated and observed physician numbers in 1998, 2000, 2002, 2004, 2006, and 2008. Our method was to compare the simulation data and observed historic data and calculate the relative and mean squared errors of the number of physicians:

$$ e=\frac{\left|\left(\widehat{y_t}-{y}_t\right)\right|}{y_t} $$

$$ \mathrm{RMSE}=\sqrt{\frac{\sum_1^n{e}_t^2}{n}} $$

Here, *y*_*t*_ represents the observed number of physicians in the year *t*; $\widehat{y_t}$represents the simulated number of physicians in the year *t*; *e* is the relative error of the number of physicians, and root mean squared error (RMSE) is the mean of the squares of the relative errors. We determined that the model was accurate if the RMSE was below 0.1, following previous study [5, 26]. We also confirmed that the RMSE did not affect indicators for evaluating the supply–demand balance.

#### Forecasting and evaluation by SD modeling and GIS

Following the validity test, we forecasted the future number of physicians in Hokkaido and each of its SMSAs. We expressed this in terms of physicians per 100 000 residents. Then, using the required numbers of physicians reported by MHLW [28], we calculated the sufficiency level defined in our previous study [5], as the indicator for evaluating the sufficiency of physician numbers:

$$ \mathrm{S}\mathrm{ufficiency}\  \mathrm{level}=\frac{\mathrm{Forecasted}\  \mathrm{number}\  \mathrm{of}\  \mathrm{physicians}}{\mathrm{Required}\  \mathrm{number}\  \mathrm{of}\  \mathrm{physicians}}\times \mathrm{Correction}\  \mathrm{coefficient} $$

We judged the sufficiency level of 1 or more to be sufficient in the target region. A sufficiency level of less than 1 was considered a shortage. In the above equation, the required physician number is derived from the claims of hospital administrators and does not include clinics. The corrective coefficient corrects for missing clinic data and is the ratio of physicians who work in a hospital to those who work in a clinic.

Using the above process, future indicators for each region were obtained by using GIS software, visualizing the forecast results on a map.

#### Evaluating future unequal distribution of physicians

To determine whether the distribution of healthcare resources was equitable, we employed the Gini coefficient to measure regional disparities [29]. The Gini coefficient, a measure of the degree of unequal distribution, is derived from the Lorenz curve, a measure of income distribution. The Gini coefficient has been employed in this manner previously. Kobayashi & Takaki used the Gini coefficient to evaluate disparities in the geographical distribution of physicians in Japan [30]. Even after this, Gini coefficient was used as indicator to evaluate quantitatively regional maldistribution of physicians [19, 31, 32]. To evaluate regional maldistribution of physicians in the future, we redefine the Gini coefficient as described below:

$$ \mathrm{Gini}\  \mathrm{coefficient}=\frac{1}{2{n}^2\mu}\sum_{i=1}^n\sum_{j=1}^n\left|{y}_i-{y}_j\right| $$

*y*_*i*_, *y*_*j*_: the number of physicians per 100 000 residents in each SMSA.

*n*: number of SMSAs.

*μ*: average of the number of physicians per 100 000 residents.

The Gini coefficient ranges from 0 to 1. In general, the smaller the value, the smaller the inequality. In our study, the smaller the value of the Gini coefficient, the less regional maldistribution.

## Results

**Fig. 4:**
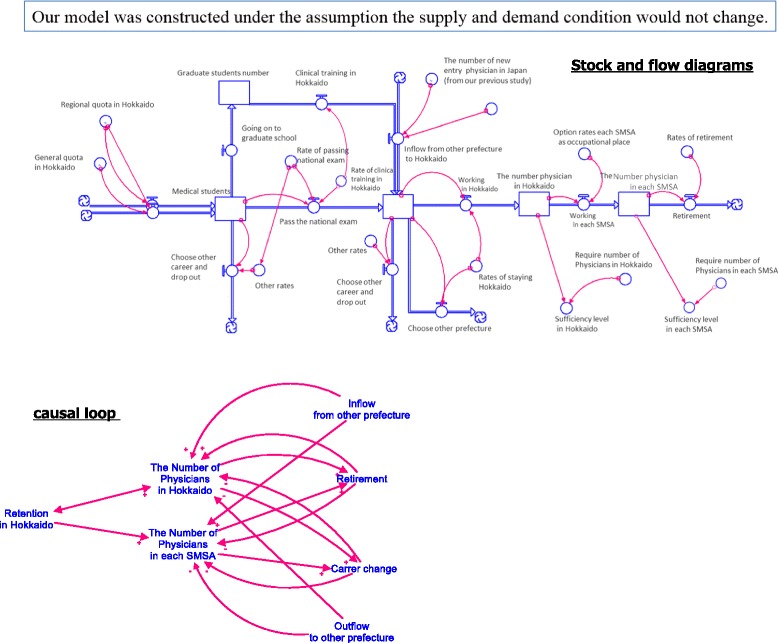
Stock and flow diagram of forecasting model based on career path

Figure 4 displays the causal loop, and stock and flow diagrams of the SD model. In this diagram, “other rates” mean rate of deviation career path of measuring object, assuming historical change include mortality, drop out, emigration rate, and so on. The model calculated the RMSE for the past 20 years as 0.002. Because the RMSE was less than 0.1, we regard this model as accurate for our research. Furthermore, we recognized that the RMSE had no effect on the sufficiency rate we defined, as discussed below. Simulated and observed historical data for specific years were compared to validate the SD model. Table 2 presents this comparison as well as the calculated RMSE.

**Table 2:** The comparison of historic data and simulation data

Year	1998	2000	2002	2004	2006	2008	MSE
Historic data	10 519	10 921	11 228	11 490	11 579	11 830	0.0245
Simulation data	10 586	10 701	11 094	11 523	11 947	12 362	
Relative error	0.000	0.000	0.000	0.000	0.001	0.002	

Table 3 shows the forecasting results for future numbers of physicians and sufficiency level in Hokkaido overall. We forecasted the number of physicians throughout the prefecture to grow from 12 019 in 2010 to 15 449 in 2030, a 28.5% increase. In addition, the number of physicians per 100 000 residents was forecasted to grow from 218.3 in 2010 to 327.4 in 2030, a 50.0% increase. The sufficiency level in Hokkaido overall was less than 1.0, indicating a shortage. However, the sufficiency level is forecasted to increase over time, reaching 1.0 in 2020. According to the forecast, the shortage will be resolved by 2020, and the trend continues throughout the span of the analysis.

**Table 3:** Forecasted results for 20 years to come

Variables	Base year	Projected				% change from 2010 to 2040
	2010	2015	2020	2025	2030	
Physician numbers in Hokkaido	12 019	12 684	13 650	14 575	15 449	28.5%
Sufficiency level in Hokkaido	0.88	0.93	1.00	1.06	1.13	28.4%
Physician numbers per 100 000 populations in Hokkaido	218.27	236.58	263.61	293.86	327.37	50.0%
Gini coefficient	0.140	0.139	0.132	0.125	0.121	−13.6%

**Fig. 5:**
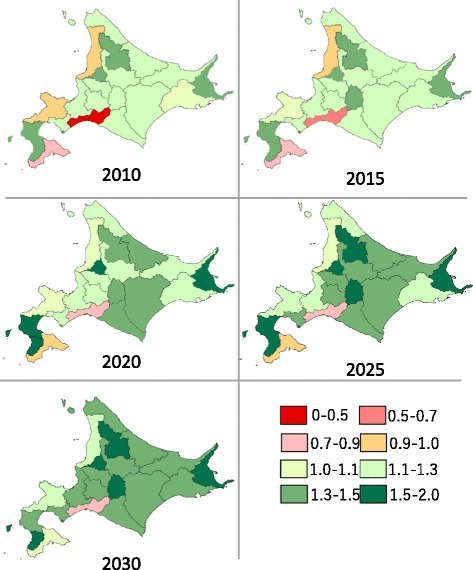
Visualizing the forecasted sufficiency level by SMSA

Figure 5 displays sufficiency levels by SMSA. Areas in red indicate shortages, while areas in green indicate sufficiency. In 2010, four secondary medical care areas were determined to have physician shortages: Minami Oshima, Shiribeshi, Higashi Iburi, and Rumoi. With the forecasted improvement in the sufficiency level throughout Hokkaido over the next 20 years, we expect only Higashi Iburi to have a shortage in 2030.

Table 3 also shows the change in the Gini coefficient value for the number of physicians forecasted for each SMSA. The value in 2010 was 0.140 and is expected to decrease to 0.121 by 2030.

## Discussion

In our study, we constructed a model to forecast the number of physicians in Hokkaido overall and in each of its SMSAs, to evaluate the number of practicing physicians in the prefecture and assess imbalances in the distribution of physicians among regions. We set up the model in consideration of the regional medical school entrance examinations and the increasing numbers of students in regional quota programs. We then tested the validity of our model by calculating the RMSE; an RMSE value of 0.l or below indicates a high degree of accuracy for the model. Generally, RMSE < 0.1 is considered reasonable [18]. Our RMSE was calculated as 0.002 < 0.1 (Table 2). Furthermore, the relative error had no effect on our evaluation of the number of physicians. We evaluated the impact of relative error as being very low and therefore concluded that our model was valid.

According to the forecast, the physician shortage in Hokkaido would be eliminated by 2019. We also forecasted that physician sufficiency levels would rise above equilibrium in all SMSAs over the forecast period. Finally, we forecasted that imbalances among regions in the distribution of physicians would diminish, as calculated by the Gini coefficient.

Looking at the sufficiency level for each SMSA in Fig. 5, we can see that some areas would still experience physician shortages after 2019, the year that we forecast the sufficiency level for physicians to reach equilibrium for Hokkaido as a whole; some regions could still experience shortages in 2030. We can therefore conclude that regional maldistribution of physicians would still be a consideration in 2019 and beyond. We propose the possibility that diminishing imbalances between SMSAs would not necessarily mean that regional maldistribution would be eliminated, as seen from the sufficiency levels of the various SMSAs.

We think that a more practical way of reducing maldistribution would be to establish a regional quota system of smaller practice areas. The current regional quota system focuses on the major areas. In the present study, the evaluation of sufficiency levels by SMSA indicates that areas of sufficiency and shortages currently coexist in the northern and eastern parts of Hokkaido Prefecture. In our viewpoint, therefore, urgent steps should be taken to place doctors in areas where our forecasting model forecasts that physician shortages could occur in the future.

Now, the Gini coefficient is becoming regarded as the gold standard for evaluation of disparity in analysis of distribution healthcare resource [29]. However, Gini coefficient is a relative value, and it is impossible to absolutely evaluate uneven distribution only from this value. Therefore, in this study, we used the Gini coefficient as an index to evaluate the tendency of uneven distribution. As it were, we evaluated whether the allocation is improving or worsening from the Gini coefficient increases or decreases. Instead of using the Gini coefficient as the sole metric for evaluating the regional distribution of physicians, we prefer to add an examination of sufficiency levels. We can evaluate regional maldistribution by comparing the sufficiency levels for a geographic unit as a whole with those of each of its constituent regions.

Some issues and limitations should be noted. A careful scrutiny of the metric for expressing adequacy of physician numbers is essential. The MHLW’s required number of physicians that we used in our research was obtained from healthcare facilities’ survey responses. This metric is therefore based on the needs of healthcare facilities and does not necessarily reflect the healthcare needs of the people. Our study’s sufficiency level metric was premised on the assumption that our survey results reflected the healthcare facilities’ grasp of the local population’s healthcare needs. In other words, we assume the required number reflect health statistics, morbidity rate, prevalence rate, and so on, based on their honest answers. The limitation is that our model assumed that the current medical care system would remain in place. This limitation gives some issues to this research. First, the criterion cannot take account of the impact of dynamic change on need. This is considered to be one of the problems in this research. Generally, as time goes on, it is expected that the required number of physicians will change. We need to account for this change when estimating the sufficient number of physicians needed in the next study. Second, there is no legal control on regional distribution of physicians in Japan. Although the number of beds or patients, the population of the area are reported as factors attracting physicians [33, 34], we do not consider these variables in the model of our study. This is because we assume that the medical environment is unchanged. However, it can be said that improvement is necessary for a more reasonable forecasting. If there are variables that can be grasped by the survey, it can be forecasted with high relevance by incorporating them into the model.

We can hypothesize that a change in the supply system for physicians, as manifested by changes in physicians’ career paths, would reduce the accuracy of our analytical results. In addition, changes in local policies, such as healthcare facility planning or a reorganization of municipalities, could alter sufficiency levels and modify the value of the Gini coefficient. If such a change in healthcare facility planning or regional policy were to take place, our model would need to be redesigned to reflect the new conditions.

In addition, this model was developed to forecast physician number in each SMSA in Hokkaido, Japan. Therefore, this is considered to be a model applicable only in Japan from the viewpoint of medical law, institution, and providing system. The modeling methodology using system dynamics for forecasting the number of doctors can also be applied in other countries. When this model is used for other countries, especially developing countries, it is necessary to reflect the difference in the medical environment in model variables.

SD modeling enabled us to analyze and estimate from multiple perspectives, considering the causal links, factor uncertainties, and different scenarios. Our SD model would be applicable to scenario analysis and sensitivity analysis. However, as mentioned above, this model cannot take account for change in medical demand for the future. To make proposal policies on human resources, analysis considering changes in demand is essential. In our next study, we plan to evaluate the supply–demand balance comprehensively by constructing an evaluation model of demand, and to combine it with this model. Increasing the collection of data on physician supply and demand, such as demographic projection and epidemiological indicators, would help construction of a reasonable supply and demand forecasting model.

## Conclusion

In this study, we focused on regional imbalances in the number of practicing physicians. These imbalances in physician numbers can lead to imbalances in the types of medical specialists available. We envision designing a model that takes this into account. Combining our research on forecasting overall physician numbers with a forecast of the number of specialists would enable us to propose a plan for training physicians that considers geographic requirements. We therefore believe that constructing a model that considers imbalances in the distribution of medical specialists is a worthwhile endeavor.
